# Factors impacting new bone formation in transcrestal sinus floor elevation followed by implant placement: a cross-sectional study

**DOI:** 10.1186/s12903-022-02352-6

**Published:** 2022-07-31

**Authors:** Zhe-Zhen Lin, Dong-Qian Xu, Yong Wang, Xue Gao, Qi Cai, Xi Ding

**Affiliations:** 1 Department of Stomatology, The First Affiliated Hospital of Wenzhou Medical University, Nanbaixiang Ouhai District, Wenzhou, 325000 Zhejiang People’s Republic of China; 2 Department of Stomatology, The Second Affiliated Hospital of Jiaxing Universtiy, 1518 Huancheng North Road, Jiaxing, 314000 Zhejiang People’s Republic of China

**Keywords:** Transcrestal sinus floor elevation, New bone formation, Dental implants, Cross-sectional study

## Abstract

**Objectives:**

This study aimed to evaluate factors related to new bone formation (NBF) following simultaneous implant placement with transcrestal sinus floor elevation (TSFE).

**Materials and methods:**

Between 2008 and 2020, 357 implants (276 patients) were placed with TSFE. Clinical and radiographic examinations were performed at the preoperative, postoperative, restoration, and follow-up stages. Marginal bone loss, during healing, and the survival rate were retrospectively analyzed.

**Results:**

Implant protrusion lengths (IPL: 3–5 mm) significantly influenced NBF during the healing period (*P-value* = 0.026, Odds Ratio = 1.15, 95% confidence interval = 1.02- 1.30). Bone grafting was correlated with NBF (*P-value* = 0.001). The distance between the implant and lateral wall of the sinus (mesial: *P-value* = 0.041, distal: *P-value* = 0.019, buccal: *P-value* = 0.032, lingual: *P-value* = 0.043) and angle between the implant and sinus floor significantly influenced NBF in four directions (mesial: *P-value* = 0.041, distal: *P-value* = 0.02, buccal: *P-value* = 0.047, lingual: *P-value* = 0.005). Implant shape (cylindrical or conical), perforations, smoking, and diabetes did not significantly affect NBF during the healing period (*P* > 0.05).

**Conclusion:**

Increasing the distance and angle between the implant and lateral wall of the sinus floor corresponded with reduced NBF. IPL may be an important factor that should be considered.

**Clinical relevance:**

Our study analyzed new bone formation following transcrestal sinus floor elevation among patients who underwent this procedure with simultaneous implant placement, several factors (including angle and distance between sinus and lateral wall and implant protrusion length) were included in our study.

**Supplementary Information:**

The online version contains supplementary material available at 10.1186/s12903-022-02352-6.

## Introduction

Transcrestal sinus floor elevation (TSFE), a procedure introduced by Tatum and Summers [1, 2], is generally accepted as an effective method for treating atrophic posterior maxillae. Considering that the Schneiderian membrane has the ability to exhibit self-osteogenesis, its osteogenic height is widely thought to be a relevant factor. New bone formation (NBF) upon TSFE is considered to be an important factor to assess clinical success of the procedure. There are many factors affecting NBF following TSFE, such as smoking, bone grafting, perforation, implant diameter, implant protrusion length (IPL), etc.

Several studies concluded that sex and age do not significantly influence NBF, and that IPL is of great significance to NBF [3–5]. Smoking, implant shape, and bone grafting are controversial factors regarding NBF that warrant further discussion. Cigarette smoking can cause hemodynamic changes, which influence the healing of implants [6]. However, several researchers have found that smoking may not affect NBF (Franceschetti et al. 2014) [7]. With regard to bone grafting, Yan M et al. (2018) found that NBF was similar between the grafting and non-grafting groups in their study [8]. Nowadays, a non-grafting procedure is being advocated for indirect sinus lift if it is found that grafting can be avoided [9]. Furthermore, the technique has been observed to reduce unnecessary trauma and expenditure. Spinell et al. (2016) discovered that TSFE without grafting could give rise to predictable NBF [10].

In addition, the sinus width is an essential factor that should be considered. Many authors speculated that a narrow sinus may promote NBF. Consequently, a wider sinus should mean less NBF compared with a narrow sinus [11, 12].

The aim of our research was to assess the factors that influence NBF in the case of simultaneous implant placement in combination with TSFE. Additionally, we aimed to investigate the survival rate and marginal bone loss (MBL).

## Methods

A total of 357 implants were placed simultaneously following TSFE in 276 patients between September 2008 and January 2020. This retrospective study was approved by the ethics committee of the First Affiliated of Wenzhou Medical University (Number 039 in 2021, 7th February in 2021) and conforms to the Declaration of Helsinki. All participants (145 men and 131 women) signed an informed consent form. Patients’ age ranged from 18 to 75 years (mean 52.5 ± 13.4), and all of them underwent thorough clinical and radiologic examinations. The study was conducted according to the Strengthening the Reporting of Observational Studies in Epidemiology (STROBE) guidelines (see Additional file 1).

### Inclusion and exclusion criteria

Patients were recruited for participation in this study based on the following criteria:

1. Required rehabilitation in the posterior maxilla
2. Attended follow-up visits for a minimum of 3 months
3. Received oral hygiene guidance and periodontal treatment
4. Controlled systemic diseases (diabetes mellitus, periodontal disease, etc.)

Patients were excluded from this study based on the following criteria:

1. Recently underwent radiation in the head and neck regions
2. Allergic to the implant material
3. Aggressive periodontitis
4. Insufficient vertical distance between the alveolar crest and occlusal plane
5. Acute sinusitis (sinusitis should be controlled)

### Surgery and prosthetic process

Upper respiratory tract infections were avoided before surgery. Under local anesthesia (Articaine Hydrochloride and Epinephrine Tartrate Injection, 4% articaine with epinephrine 1:100,000, PRODUITS DENTAIRES PIERRE ROLLAND, France), a midcrestal incision above the alveolar crest was performed to elevate a flap. Every implant site was prepared to a depth of 1–2 mm below the sinus floor by sequential drilling. Several consecutive modified osteotomes, introduced by Summers, was used to elevate the sinus floor to achieve the final depth. Perforation was discovered according to clinical, and tactile assessment, combined with the radiographic and clinical appearance. Implants were inserted, healing abutments or cover screws were installed, then the flap was sutured tightly. Postoperatively, antibiotics were prescribed with Cephalosporins (Cefaclor Sustained Release Tablets, Suzhou sikro Pharmaceutical Co., Ltd, China, 750 mg each time, twice a day) and tinidazole (Tinidazole Capsules, Jiangsu Changjiang Pharmaceutical Co., Ltd, China, 1 g each time, once a day) for 4 days, gargle with chlorhexidine for 4–5 days (Jiangsu Zhiyuan Pharmaceutical Co., Ltd, three times a day). Oral hygiene was performed as normal. Sutures were removed after 7–14 days. Smoking, swimming, sniffing, and traveling by plane were forbidden before sutures removal. Implant-supported fixed dental prostheses or single crowns were delivered to patients after a 3–8-month healing period. NBF was measured after the healing period.

### Details of study

Of the 357 implants, 215 implants were in replacement of the first molar, accounting for more than half of the total number of implants. The other implant sites included the second molar (n = 84), second premolar (n = 50), and first premolar (n = 8). The implant diameters of the 357 implants placed in this study varied from 3.3 mm to 5.0 mm, and the implant types consisted of Straumann® implant systems (3.3 mm, 4.1 mm, 4.8 mm) and Nobel® implant systems (4.3 mm, 5.0 mm). Further, the length of the selected implants ranged from 8 to 12 mm, and it should be mentioned that 61 short implants (length = 8 mm) were placed in our study owing to severe bone atrophy. During the surgery, 50 implants (35 patients) were placed along with bone substitutes (Geistlich Bio-Oss), and 36 minor lacerations were discovered in the Schneiderian membrane. If perforations occurred, patients were told not to sniff and swim.

**Fig. 1:**
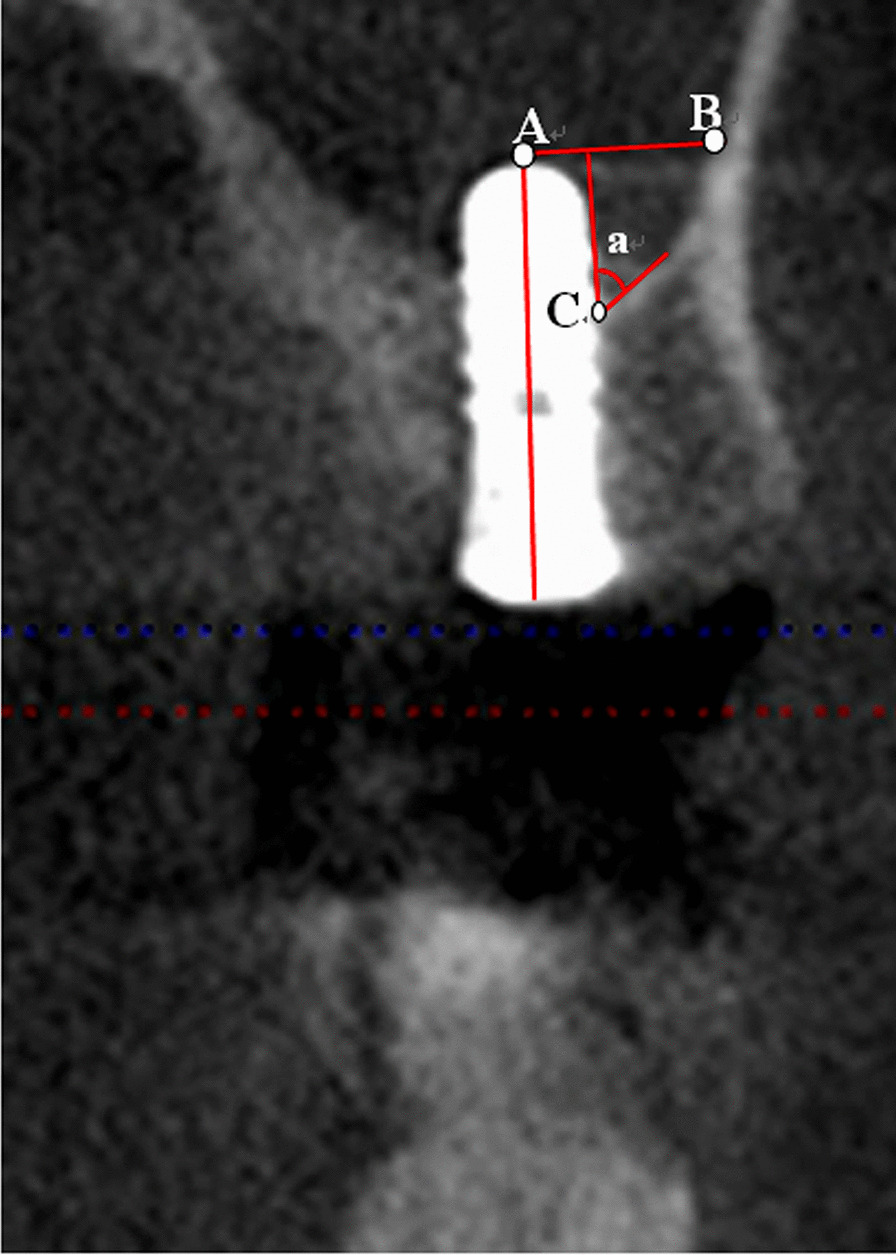
The distance between the implant and sinus floor (the distance between **A** and **B**) and angle between the implant and sinus floor (∠a). Point **A**: The highest point where the implant enters the sinus. Line AB: In relation to Point A, we drew a vertical line along the long axis of the implant. Point **B**: The vertical line intersects with the lateral sinus floor. ∠a: The angle between the implant margin and bone slope of the lateral sinus floor

When implants were inserted and restoration had occurred, NBF was measured through cone-beam computed tomography (CT, KaVo X-Trend). Only high-quality images were included in the analysis of NBF and MBL. The number of high-quality images for NBF was 107, and the number of high-quality images for MBL was 193. The failed implants were not included in the analysis of NBF. The relationship between NBF and several factors (implant protrusion length, the location of implant sites, bone grafting, perforation, smoking, implant shape, and implant diameter) were investigated in this study. The distance (Fig. 1: Point A: The highest point where the implant enters the sinus. Line AB: In relation to Point A, a vertical line was drawn along the long axis of the implant. Point B: The vertical line intersects with the lateral floor of the sinus) between the implant and sinus floor and angle (Fig. 1: ∠a, the angle between the implant margin and bone slope of the lateral sinus floor) between the implant and sinus floor were measured, and their correlation with NBF was evaluated.

Implant survival was determined based on the following criteria outlined at the Pisa Consensus Conference: [13] (1) No pain during function, (2) No mobility, (3) Remains in the mouth, and (4) No controlled exudate. If an implant did not meet the above mentioned criteria, it was considered a failed implant that had to be removed, and the patient received another implant after a healing period of a minimum of 3 months.

### Statistical analysis

The linear generalized estimating equation model (GEE) was performed to analyze the resulting NBF (bone grafting, perforation, smoking status, implant shape, number of implants, implant protrusion length, implant diameter) and MBL, and binary was used for the survival rate during the healing period, Odds Ratio (OR) and confidence interval (CI) was used to assess these two variables. And multivariate analysis was used for angle and distance between implant and sinus floor (see Additional Files 2). The statistical analysis was performed using IBM SPSS Statistics 17.0 (Chicago, USA) and Excel (Microsoft 2010).

## Results

The mean residual bone height at the treated sites was 7.02 ± 1.64 mm. Ten of the 357 implants failed during the healing period, and three implants failed during the 1-year loading period. The survival rate during the healing period of these implants was 96.4%, and cylindrical implants exhibited a survival rate of 97.2%, while conical implants exhibited a survival rate of 97.0% (*P*-value = 0.736, OR = 1.32, 95% CI: 0.26–6.58). When the IPL was above 3 mm, the survival rate during the healing period (94.3%) was lower (*P*-value = 0.029, OR = 5.80, 95%CI: 1.20–28.05). As shown in Table 2, the IPL was the key factor that affected the survival rate during the healing period. Meanwhile, perforation and grafting had no significant influence on the survival rate during the healing period.

### New bone formation

**Fig. 2:**
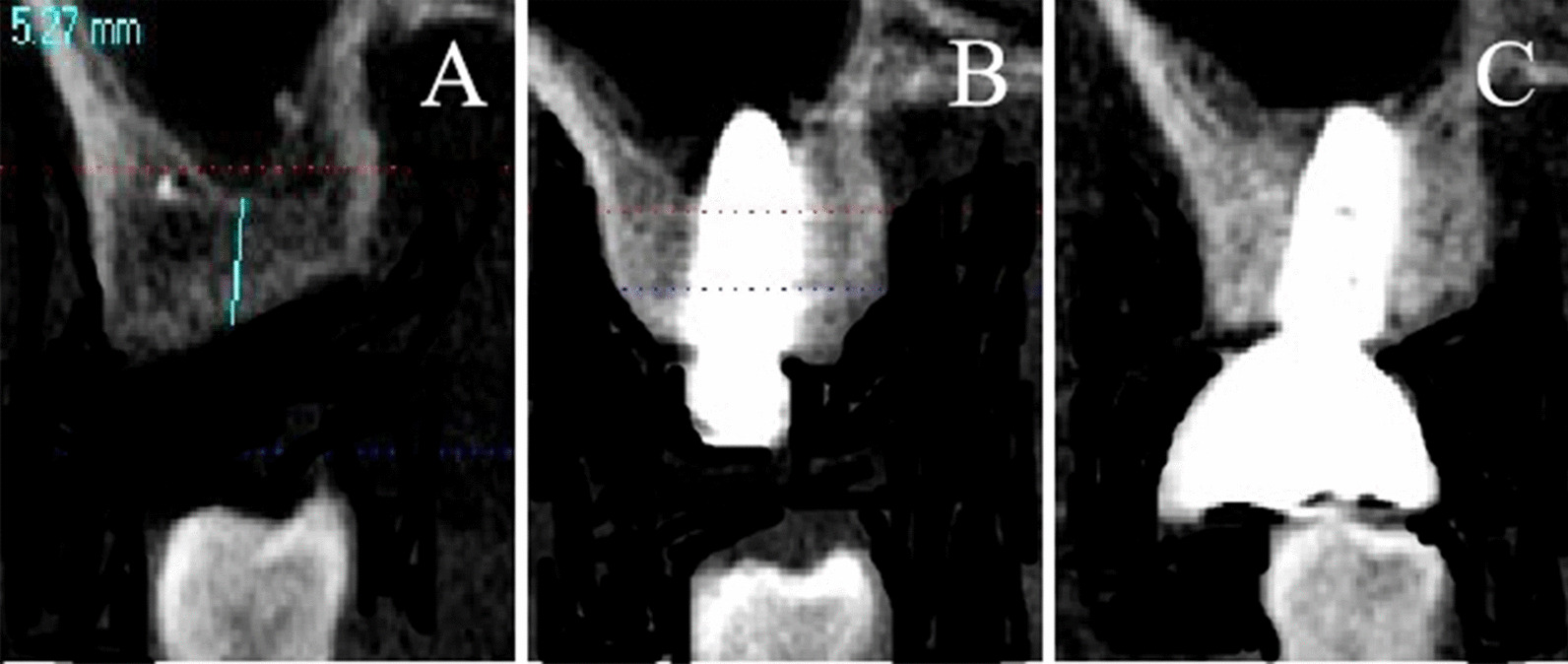
**A-C** An implant inserted into the sinus floor resulting in a large amount of new bone formation with a relatively small angle and distance between the implant and sinus floor (**A**: before the surgery, **B**: after the surgery, **C**: after the restoration)

**Fig. 3:**
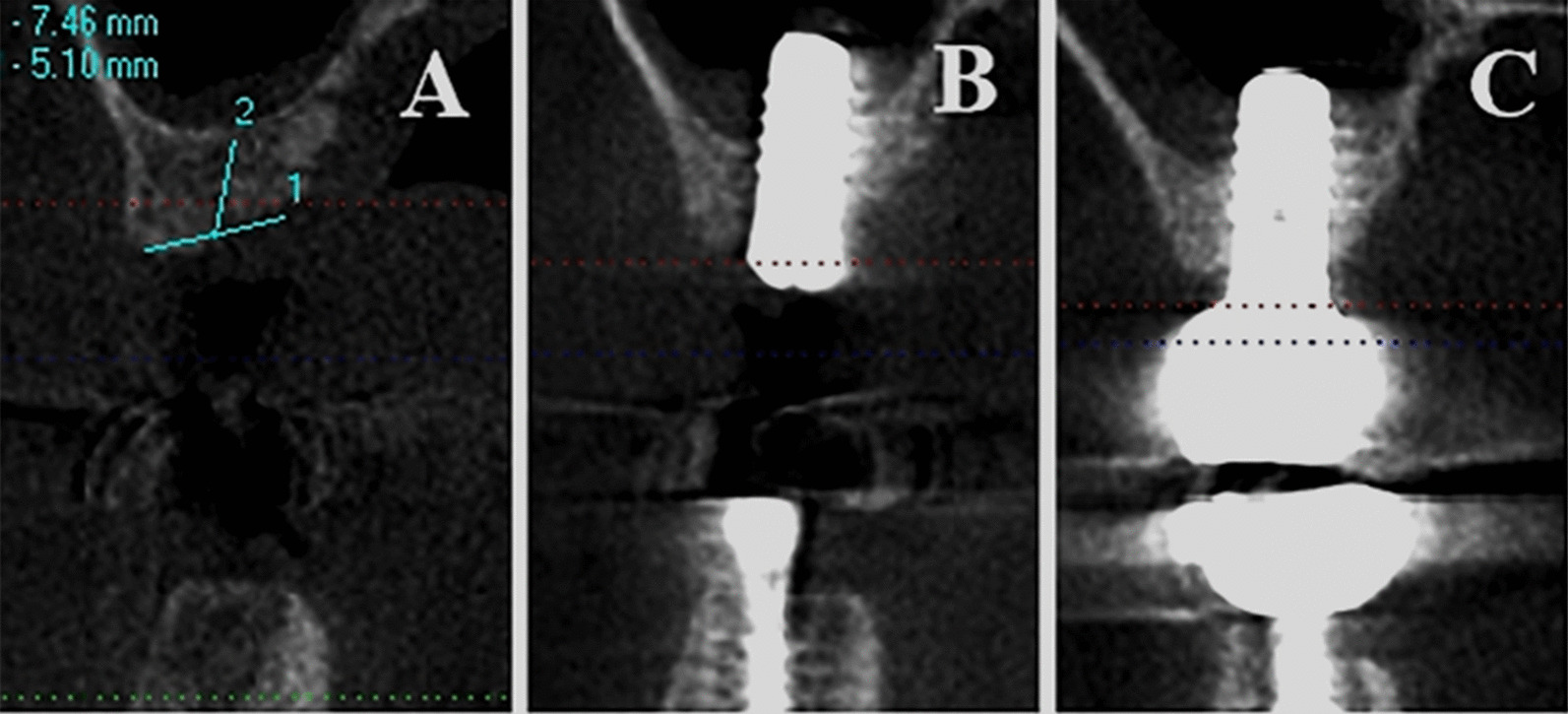
**A-C** An implant inserted into the sinus floor resulting in a small amount of new bone formation. The adjacent teeth are natural teeth or implants that were placed without transcrestal sinus floor elevation. The angle and distance between the implant and sinus floor are relatively large (**A**: before the surgery, **B**: after the surgery, **C**: after the restoration)

As shown in Table 1, similar results concerning NBF during the healing period were discovered in four directions (lingual, buccal, distal, mesial); the distance and angle between the sinus floor and implant had a significant influence on NBF in the lingual (distance: *P-value* = 0.043, angle: *P-value* = 0.005), buccal (distance: *P-value* = 0.032, angle: *P-value* = 0.047), distal (distance: *P-value* = 0.019, angle: *P-value* = 0.02), and mesial (distance: *P-value* = 0.041, angle: *P-value* = 0.041) directions. Shorter distance between the sinus floor and implant corresponded with greater NBF (Figs. 2 and 3). Furthermore, smaller angles between the implant and sinus floor, also corresponded with greater NBF.

**Table 1:** Univariate analysis of new bone formation during the healing period (in four directions)

	Mesial	Distal	Buccal	Lingual
Distance (mm)	4.19 ± 1.00	4.56 ± 1.14	4.65 ± 1.29	4.51 ± 1.24
*P*-value	0.041	0.019	0.032	0.043
Angle	49.00 ± 9.49	49.40 ± 8.45	48.14 ± 11.00	47.25 ± 11.17
*P*-value	0.041	0.020	0.047	0.005
Bone formation (mm)	1.57 ± 0.97	1.53 ± 1.00	1.62 ± 1.05	1.62 ± 1.01

Distance: Distance between the sinus floor and implant (Fig. 1: distance between A and B)

Angle: ∠a: The angle between the implant margin and bone slope of the lateral sinus floor

(Fig. 1: ∠a)

**Fig. 4:**
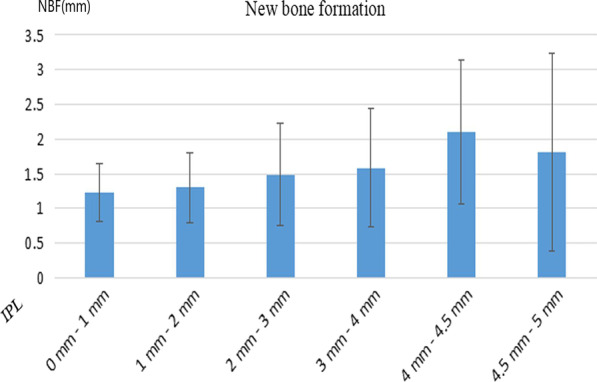
The relationship between implant protrusion length (IPL) and new bone formation (NBF)

As summarized in Tables 2 and 3, the IPL is a key factor related to NBF; IPL that did not exceed 5 mm had a significant positive influence on NBF during the healing period (NBF: IPL ≥ 3 mm: 1.74 ± 1.12 mm, IPL < 3 mm: 1.36 ± 0.61 mm, univariate analysis: *P-value* = 0.03, multivariate analysis: *P-value* = 0.026, OR = 1.15, 95% CI: 1.02–1.30). Figure 4 illustrates the relationship between IPL and NBF during the healing period. In these cases of IPL between 4 mm and 4.5 mm, the largest NBF was obtained during the healing period. Another important factor was bone substitutes; the sites at which bone substitutes were used exhibited 2.22 ± 1.13 mm of NBF during the healing period, which was relatively higher than that at non-grafting sites (1.43 ± 0.79 mm, univariate analysis: *P-value* = 0.001, multivariate analysis: *P-value* = 0.001, OR = 0.28, 95%CI: 0.13–0.57).

**Table 2:** Multivariate analysis of new bone formation in transcrestal sinus floor elevation

Factor	New bone formation	*P*-value
*Bone grafting*		
Yes	2.77 ± 1.35 mm	0.001
No	1.43 ± 0.79 mm	
*Perforation*		
Yes	1.34 ± 0.91 mm	0.284
No	1.62 ± 0.96 mm	
*Smoking status*		
Smoker	1.68 ± 0.85 mm	0.684
Non-smoker	1.57 ± 0.98 mm	
*Implant shape*		
Cylindrical	1.68 ± 1.01 mm	0.200
Conical	1.41 ± 0.83 mm	
*Number of implants*		
Single	1.53 ± 0.98 mm	0.478
Double and adjacent	1.70 ± 0.91 mm	
*Implant protrusion length*		
3–5 mm	1.74 ± 1.12 mm	0.030
0–3 mm	1.36 ± 0.61 mm	
*Implant diameter*		
≤ 4.3 mm	1.61 ± 1.15 mm	0.899
≥ 4.8 mm	1.58 ± 0.91 mm

**Table 3:** Multivariate analysis of new bone formation during the healing period

Factor	OR	95% confidence interval	*P*-value
Perforation	1.49	1.00–2.23	0.053
Bone grafting	0.28	0.13–0.57	0.001
Implant protrusion length	1.15	1.02–1.30	0.026
Location of treatment sites	0.87	0.57–1.34	0.537
Implant shape	1.41	0.97–2.05	0.076

**Fig. 5:**
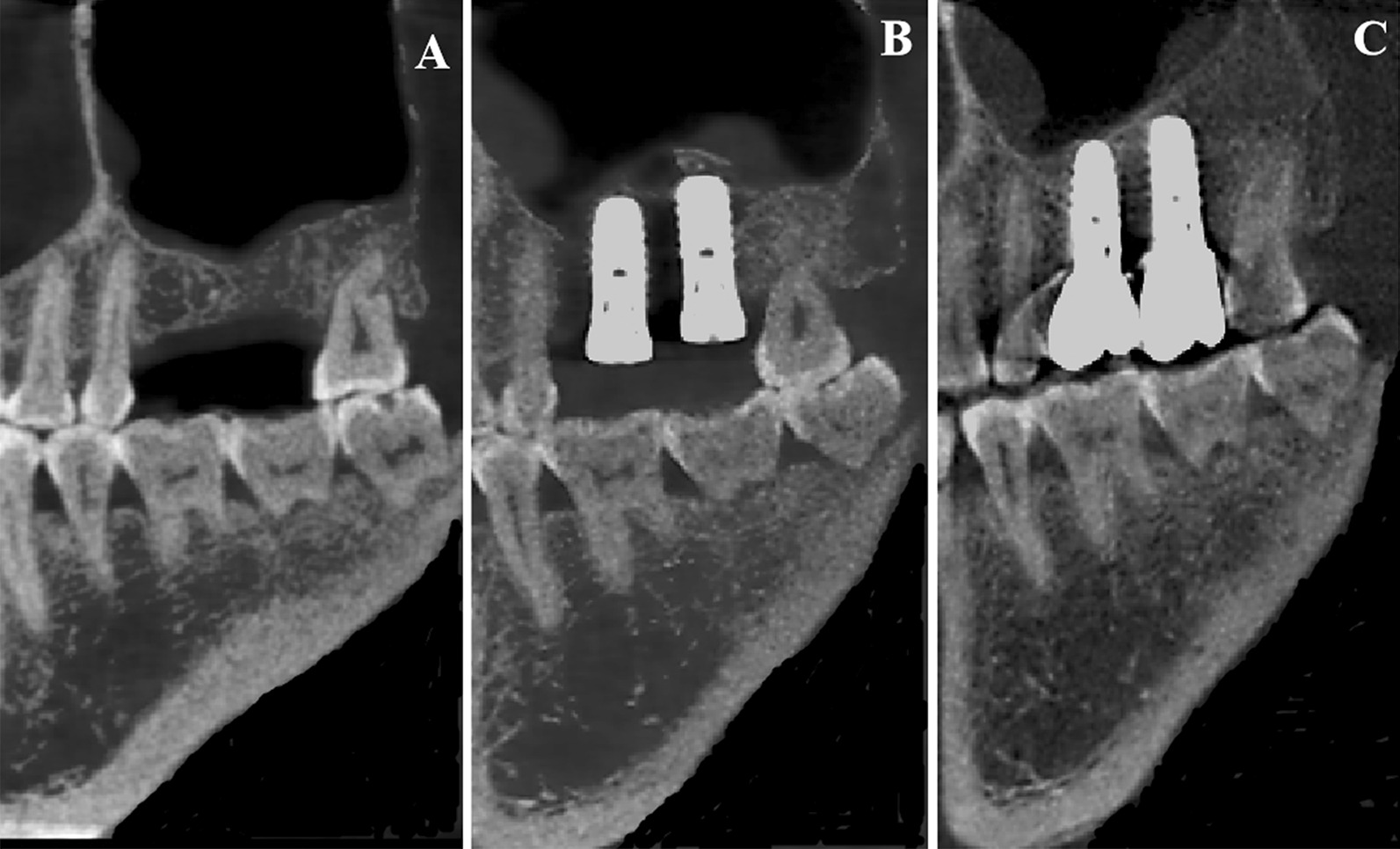
A patient who underwent transcrestal sinus floor elevation at two sites, resulting in satisfactory new bone formation. The angle and distance between the implant and lateral wall of the sinus are small. (**A**: before the surgery, **B**: after the surgery, **C**: after the restoration)

NBF associated with adjacent implants that were placed following TSFE (1.70 ± 0.91 mm) was greater than that at a single implant site (1.53 ± 0.98 mm, *P-value* = 0.478, single sites: the adjacent teeth were natural teeth or implants that were not placed with TSFE (Fig. 5). Further, the NBF at perforated sites was 1.34 ± 0.91 mm, and non-perforated sites exhibited 1.62 ± 0.96 mm (*P-value* = 0.284). Smokers (1.68 ± 0.85 mm) did not exhibit lower NBF compared with non-smokers (1.57 ± 0.98 mm, *P-value* = 0.684). Meanwhile, cylindrical implants (1.68 ± 1.01 mm) did not significantly exhibited greater NBF than conical implants (1.41 ± 0.83 mm, *P-value* = 0.20). Wide implants resulted in similar NBF to that of narrow implants (*P-value* = 0.899). Table 3 summarizes results of univariate analyses, according to which the aforementioned five factors did not result in significant differences in NBF during the healing period.

Table 3 also illustrates that perforation (*P-value* = 0.053, OR = 1.49, 95%CI: 1.00–2.23), implant shape (*P-value* = 0.076, OR = 1.41, 95%CI: 0.97–2.05), and the location of the treatment sites (*P-value* = 0.537, OR = 0.87, 95%CI: 0.57–1.34) had no obvious influence on NBF based on the results of the multivariate analysis.

### Marginal bone loss

As shown in Table 4, smoking and diabetes did not have a significant influence on MBL during the healing period (smoking: OR = 0.99, 95%CI: 0.92–1.07, *P-value* = 0.803, diabetes: OR = 1.01, 95%CI: 0.93–1.10, *P-value* = 0.751). Furthermore, the multivariate analysis revealed another four factors (perforation: *P-value* = 0.165, bone grafting: *P-value* = 0.77, healing method: *P-value* = 0.738, and implant shape: *P-value* = 0.264) that did not influence the MBL during the healing period.

**Table 4:** Multivariate analysis of marginal bone loss during the healing period

Factor	Odds ratio	95% confidence interval	*P*-value
Perforation	1.05	0.98–1.14	0.165
Bone grafting	0.99	0.92–1.06	0.770
Diabetes	1.01	0.93–1.10	0.751
Smoking	0.99	0.92–1.07	0.803
Implant shape	1.04	0.97–1.11	0.264
Healing method	0.99	0.94–1.05	0.738

## Discussion

This retrospective study assessed significant factors that were critical to NBF in TSFE. NBF following TSFE has been discussed in many reports [12, 14]; these studies concentrated on histomorphometric methods, and our study adopted radiographic methods to confirm conclusions. Smoking, bone grafting, perforation, implant shape, implant diameter, IPL, and the distance and angle between sinus floor and implant were analyzed in our study.

Most importantly, our findings confirmed that if the distance and angle between the implant and sinus floor were large, the bone-implant contact area would be small, which would lead to a reduced possibility of osteogenesis [15]. We analyzed NBF in four directions (mesial, distal, buccal, and lingual). Our findings therefore not only confirm previous findings but also present novel results. Furthermore, several studies have indicated that the width of the sinus might influence NBF [11, 12]. A wider sinus meant a larger distance and angle between the implant and sinus floor; our study had a similar conclusion.

The IPL appeared to be another critical factor affecting NBF. In our study, the degree of NBF was different in different IPL-based groups. When the IPL was between 3 and 5 mm, NBF was significantly greater. The result was consistent with previous studies. Several researchers have elucidated the relationship between IPL and NBF, reporting that NBF increased with an increase in IPL [3, 14, 16]. However, previous studies have seldom explained the relationship between IPL and implant survival rate. Considering the mucosa’s osteogenic ability, it can guarantee a satisfactory survival rate as long as it slightly protrudes into the maxillary sinus. Nevertheless, varying classification methods for IPL would result in different outcomes, and accidental events may lead to contradictory conclusions if the sample size is limited. It did not mean higher IPL was beneficial to clinical performance, we still strictly controlled indications.

There were few debates on the above two factors; however, bone grafting was a controversial topic for NBF. Our findings are consistent with those of Nedir et al., [17]. who suggested that bone grafting might significantly influence NBF in the short term. In our study, bone grafting was shown to increase NBF during the healing period. Significantly, the NBF and non-resorbable bone substitute were different upon cone-beam CT. However, during the long-term follow-up period, bone grafting did not affect NBF [18].

Although the number of implants that were placed with TSFE did not significantly influence NBF during the healing period, adjacent implants that were placed with TSFE exhibited greater NBF. If the sites that were subjected to TSFE were adjacent, the resulting tent shape would ensure that the sinus membrane remained in an elevated position [19]; consequently, the distance between the implants and sinus wall would be shorter, which could lead to an increase in NBF during the healing period.

Perforation is another controversial debate in TSFE. Lacerating the Schneiderian membrane is extraordinarily common in sinus lift procedures due to it not being visible to the surgeon; this should be considered important. Several doctors are accustomed to terminating the TSFE or changing it to a direct approach [20]. However, Rammelsberg et al. (2020) suggested that perforation is not directly related to implant failure, which was confirmed in our research [21]. The NBF at the perforated sites was lower than that at the non-perforated sites, but no significant difference was found between the two groups. Nevertheless, it was not possible to detect some of the perforations caused during TSFE via a clinical examination. In other words, implant failure might have occurred due to lacerating the membrane in cases in which the lacerations could not be detected. This insight should be seriously considered [22, 23].

The strength of our study is that we have investigated several factors that are related to NBF, which were seldom mentioned in previous reports. Nevertheless, there are many limitations associated with our study, which should be considered. The sample size and failed implants were rather limited in number, which could not be ignored. Taking these factors into consideration, the results should be interpreted with caution. Additionally, our study is limited due to its retrospective nature, as we only used existing information. In the future, a prospective study on NBF with a larger sample size should be conducted.

## Conclusion

Within limitations of the study, the distance and angle between the implant and sinus floor might influence NBF. Furthermore, the IPL plays a significant role, which should be considered when performing TSFE. In addition, if adjacent implants are placed with TSFE, the NBF is greater than that at a single implant site; however, no significant differences were found between these two groups in our study. Bone grafting, perforation, diabetes, smoking, and implant length do not have a significant influence on NBF.

## Supplementary Information

**Additional file 1.** Stata for survival rate during the healing period.

**Additional file 2.** Stata for new bone formation and marginal bone loss during the healing period.

## Data Availability

The data that supports the findings of this study are available in the supplementary material of this article.

## References

[1] Tatum H (1986). Maxillary and sinus implant reconstructions. Dent Clin North Am.

[2] Summers RB (1994). A new concept in maxillary implant surgery: the osteotome technique. Compendium.

[3] Si MS, Shou YW, Shi YT, Yang GL, Wang HM, He FM (2016). Long-term outcomes of osteotome sinus floor elevation without bone grafts: a clinical retrospective study of 4–9 years. Clin Oral Implants Res.

[4] Gai L, Luo X, Guan Y, He F (2021). Comparative evaluation of endo-sinus bone augmentation after Osteotome sinus floor elevation without grafting using two radiographic methods. Int J Oral Maxillofac Implants.

[5] Suk-Arj P, Wongchuensoontorn C, Taebunpakul P (2019). Evaluation of bone formation following the osteotome sinus floor elevation technique without grafting using cone beam computed tomography: a preliminary study. Int J Implant Dent.

[6] Atarchi AR, Miley DD, Omran MT, Abdulkareem AA (2020). Early failure rate and associated risk factors for dental implants placed with and without maxillary sinus augmentation: a retrospective study. Int J Oral Maxillofac Implants.

[7] Franceschetti G, Farina R, Stacchi C, Di Lenarda R, Di Raimondo R, Trombelli L (2014). Radiographic outcomes of transcrestal sinus floor elevation performed with a minimally invasive technique in smoker and non-smoker patients. Clin Oral Implants Res.

[8] Yan M, Liu R, Bai S, Wang M, Xia H, Chen J (2018). Transalveolar sinus floor lift without bone grafting in atrophic maxilla: a meta-analysis. Sci Rep.

[9] French D, Nadji N, Shariati B, Hatzimanolakis P, Larjava H (2016). Survival and success rates of dental implants placed using osteotome sinus floor elevation without added bone grafting: a retrospective study with a follow-up of up to 10 years. Int J Periodontics Restor Dent.

[10] Spinelli D, De Vico G, Condò R, Ottria L, Arcuri C (2016). Transcrestal guided sinus lift without grafting materials: a 36 months clinical prospective study. Oral Implantol Rome.

[11] Pignaton TB, Spin-Neto R, Ferreira CEA, Martinelli CB, de Oliveira GJPL, Marcantonio E (2020). Remodelling of sinus bone grafts according to the distance from the native bone: a histomorphometric analysis. Clin Oral Implants Res.

[12] Stacchi C, Lombardi T, Ottonelli R, Berton F, Perinetti G, Traini T (2018). New bone formation after transcrestal sinus floor elevation was influenced by sinus cavity dimensions: a prospective histologic and histomorphometric study. Clin Oral Implants Res.

[13] Misch CE, Perel ML, Wang HL, Sammartino G, Galindo-Moreno P, Trisi P, Steigmann M (2008). Implant success, survival, and failure: the international congress of oral implantologists (ICOI) Pisa consensus conference. Implant Dent.

[14] Yang J, Xia T, Fang J, Shi B (2018). Radiological changes associated with new bone formation following osteotome sinus floor elevation (OSFE): a retrospective study of 40 patients with 18-month follow-up. Med Sci Monit.

[15] Avila G, Wang HL, Galindo-Moreno P, Misch CE, Bagramian RA, Rudek I (2010). The influence of the bucco-palatal distance on sinus augmentation outcomes. J Periodontol.

[16] Lai HC, Zhuang LF, Lv XF, Zhang ZY, Zhang YX, Zhang ZY (2010). Osteotome sinus floor elevation with or without grafting: a preliminary clinical trial. Clin Oral Implants Res.

[17] Nedir R, Nurdin N, Khoury P, Perneger T, Hage ME, Bernard JP (2013). Osteotome sinus floor elevation with and without grafting material in the severely atrophic maxilla. A 1-year prospective randomized controlled study. Clin Oral Implants Res..

[18] Yan M, Liu R, Bai S, Wang M, Xia H, Chen J (2018). Transalveolar sinus floor lift without bone grafting in atrophic maxilla: a meta-analysis. Sci Rep.

[19] Nedir R, Nurdin N, Abi Najm S, El Hage M, Bischof M (2017). Short implants placed with or without grafting into atrophic sinuses: the 5-year results of a prospective randomized controlled study. Clin Oral Implants Res.

[20] Zill A, Precht C, Beck-Broichsitter B, Sehner S, Smeets R, Heiland M, Rendenbach C (2016). Implants inserted with graftless osteotome sinus floor elevation–A 5-year post-loading retrospective study. Eur J Oral Implantol.

[21] Rammelsberg P, Kilian S, Büsch C, Kappel S (2020). The effect of transcrestal sinus-floor elevation without graft on the long-term prognosis of maxillary implants. J Clin Periodontol.

[22] Park WB, Kang KL, Park JS, Han JY (2021). One-step lateral window approach for removal of benign minor sinus pathologies combined with transcrestal sinus floor elevation without bone grafts: a retrospective study. Int J Oral Maxillofac Surg.

[23] Park WB, Herr Y, Chung JH, Shin SI, Han JY, Lim HC (2021). Long-term effects of sinus membrane perforation on dental implants placed with transcrestal sinus floor elevation: a case-control study. Clin Implant Dent Relat Res.

